# Predicting the In Vivo Performance of Cardiovascular Biomaterials: Current Approaches In Vitro Evaluation of Blood-Biomaterial Interactions

**DOI:** 10.3390/ijms222111390

**Published:** 2021-10-21

**Authors:** Anne Strohbach, Raila Busch

**Affiliations:** 1 Department of Internal Medicine B Cardiology, University Medicine Greifswald, Ferdinand-Sauerbruch-Str., 17475 Greifswald, Germany; buschr@uni-greifswald.de; 2 DZHK (German Centre for Cardiovascular Research), Partner Site Greifswald, Fleischmannstr. 42-44, 17489 Greifswald, Germany

**Keywords:** biomaterial, blood compatibility, complement activation, coagulation, in vitro testing

## Abstract

The therapeutic efficacy of a cardiovascular device after implantation is highly dependent on the host-initiated complement and coagulation cascade. Both can eventually trigger thrombosis and inflammation. Therefore, understanding these initial responses of the body is of great importance for newly developed biomaterials. Subtle modulation of the associated biological processes could optimize clinical outcomes. However, our failure to produce truly blood compatible materials may reflect our inability to properly understand the mechanisms of thrombosis and inflammation associated with biomaterials. In vitro models mimicking these processes provide valuable insights into the mechanisms of biomaterial-induced complement activation and coagulation. Here, we review (i) the influence of biomaterials on complement and coagulation cascades, (ii) the significance of complement-coagulation interactions for the clinical success of cardiovascular implants, (iii) the modulation of complement activation by surface modifications, and (iv) in vitro testing strategies.

## 1. Introduction

The implantation of a biomaterial disturbs the hemostasis of the surrounding tissue. Consequently, the implant is exposed to blood proteins which rapidly adsorb non-specifically onto its surface [1,2]. Within minutes, the biomaterial induces a host response to the implant, which aims to isolate the foreign material from the host immune system [3]. This response can diminish the efficacy of the treatment and eventually determine functionality and clinical outcome [4,5]. In this context, the term “biocompatibility” describes “the ability of a material to perform with an appropriate host response in a specific application” [6]. Given the refined therapeutic approaches for managing biomaterial-induced adverse reactions, one could question the need to improve the biological performance of materials. The need is there, yet. Pharmacological management of material-related thrombosis is associated with a high risk of bleeding and decreases patient safety and quality of life [7].

A broad field of application of biomaterials is the use of blood-contacting cardiovascular devices such as stents, artificial heart valves, or vascular prostheses. Under physiologic conditions, blood contacts the negatively charged endothelium, which is anti-coagulant and anti-thrombotic. The implantation of a cardiovascular device represents the introduction of a foreign surface in the circulation, which does not necessarily come with these properties. Immediately after the first contact with blood, plasma proteins and interstitial fluids adsorb to the biomaterial surface. The result of such interference may be an inappropriate activation of the contact and complement systems, which trigger platelets and immune cells through pro-thrombotic and pro-inflammatory mediators, respectively [8,9,10]. However, after surface contact, complement factors stimulate a direct infiltration of immune cells to the implant region to destroy or remove the unwanted structures by phagocytosis [11]. To some extent, this host response is essential for healing after the implantation process. Systemic inflammatory activation, however, may eventually lead to implant failure [6]. Biomaterials are known to act as agonists of complement and leukocyte activation. This is frequently studied only in the context of inflammation, while most blood compatibility testing of cardiovascular devices relates to thrombotic responses induced by the biomaterial [9]. The association of the complement system and immune cells with the coagulation cascade and thrombosis is gaining increasing attention.

Cardiovascular biomaterials—metals and their oxides, polymers, pyrolytic, and diamond-like carbon—encompass a wide range of compounds, from naturally derived biological macromolecules to synthetic coatings. All of which differ in function and structural features [4]. The biomaterial can be permanent, intended to stay in the body for a lifetime, or temporary with degradation times of several months to years. Among the available polymeric cardiovascular biomaterials, polyesters are the preferred materials for manufacturing bioabsorbable stents. Poly-(l-lactic) acid (PLLA), polyglycolic acid (PGA), and poly(d,l-lactide/glycolide) copolymer (PDLA) are some of the most commonly used bioabsorbable polymers [12,13]. Current absorbable stent technologies rely on the polymer PLLA [14]. Copolymerization with small amounts of other polymers such as poly-ε-caprolactone (PCL) optimizes the physical properties [15] and hemocompatibility [16]. However, compared to other polymer materials, these polyesters show only moderate blood compatibility [13]. Many approaches aim to optimize the biological performance of polymer surfaces. Yet, a truly biocompatible material has not been identified, nor is the complex process of host response fully understood [7]. Therefore, most cardiovascular devices function with little or acceptable risk of complications. This review outlines the current state of understanding these phenomena with particular reference to polymeric biomaterials for cardiovascular devices.

## 2. Complement Activation by Biomaterials

The complement system is composed of more than 30 proteins, which are present as membrane-associated proteins or circulate in the plasma as part of the innate immune response. Activation of the complement system occurs via three different pathways: the classical pathway, the lectin pathway, and the alternative pathway (thoroughly reviewed in [17]). Complement is activated by the same stimuli that launch inflammation when the danger of infection is detected or when the host tissue is damaged [18]. These situations are frequently accompanied by activation of the coagulation system. One should consider that the complement and coagulation cascades act locally at the site of infection and of bleeding, respectively. However, the systemic activation of these cascades, e.g., by the presence of an artificial surface, might seriously threaten the host. We will, therefore, focus on the influence of biomaterials on complement activation.

**Figure 1:**
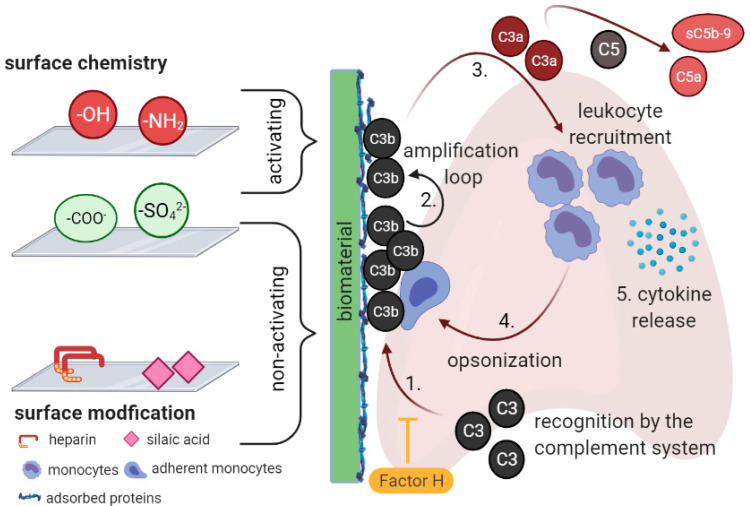
Complement activation by biomaterials through the alternative pathway. After first contact with blood, plasma proteins quickly adsorb to the biomaterial surface. The composition of the adsorbed protein layer subsequently triggers complement activation by covalent binding of C3b (1.), which activates the amplification loop, generating more C3b fragments from circulating C3 (2.). Eventually, the C3b fragments conceal the protein layer, which enhances the release of C3a, C5a, and sC5b-9 into the fluid phase. These fragments are potent chemoattractants that recruit monocytes to the biomaterial surface (3.). Active monocytes recognize surface bound C3b through CD11b/CD18 (Mac-1) and initiate opsonization (4.) and cytokine release (5.). Factor H is a major soluble inhibitor of complement activation and negatively charged surfaces accelerate its binding to immobilized C3b, thus providing a non-activating surface. Created with BioRender.com.

Complement activation by biomaterials is always associated with rapid binding of C3 to the adsorbed protein layer on the biomaterial [19,20], followed by conformational changes, which directly triggers the alternative pathway (Figure 1) [21]. Following complement activation, C3b covalently binds to the protein layer on the artificial surface which leads to the generation of more C3b fragments (amplification loop). Once the original protein layer is covered by these C3b fragments, the release of anaphylatoxins into the plasma is initiated [2]. These anaphylatoxins are potent chemoattractants that recruit leukocytes to the biomaterial, which in turn recognize surface-bound C3b fragments via ligands such as CD11b/CD18 (Mac-1) and initiate opsonization and cytokine release [17,22]. In that respect, the mechanisms of leukocyte adhesion on artificial surfaces are not yet clear. In vitro work suggests that it is mediated in part by the complement product iC3b, since inhibition of complement activation significantly reduced leukocyte adhesion [9]. Factor H regulates complement activation via C3 by inhibiting the formation of C3 convertase which catalyzes the cleavage of C3 into C3a and C3b [23]. Subsequently, C5 convertase is generated cleaving C5 in C5a—which is also an anapylatoxin—and C5b. C5b binds to the foreign surface and initiates the generation of membrane attack complex (C5b-9). As a result of this process, a series of inflammatory reactions is induced.

Biomaterials can be classified as “non-activating” or “activating” surfaces (Figure 1). Negatively charged groups such as carboxyl and sulfate, sialic acid and bound heparin seem to promote high-affinity association between bound C3b and Factor H—the major soluble inhibitor of complement—thereby providing a non-activating surface [24]. On the other hand, the presence of neutral and positively charged groups such as hydroxyl groups and amino groups activates the alternative pathway which facilitates covalent binding of C3b and therefore provides an activating microenvironment [25,26,27]. However, even in the absence of these activating groups, some biomaterials are able to activate complement suggesting that there are other mechanisms of material-induced complement activation. Of note, non-activating coatings were not found to be useful on their own, neither in cardiovascular stents nor in other devices [7,28]. There is, however, one stent available combining such a non-activating coating with drug elution. The cobalt alloy Endeavor® Resolute is coated with phosphorylcholine, which mimics the surface of cell membranes. Interestingly, this stent has recorded very low number of late stent thrombosis, which is proven in numerous clinical trials [29,30,31]. Phosphorylcholine exhibits excellent blood compatibility at a molecular level due to the biomimetic structure [32]. Despite the numerous advantages, current phosphorylcholine-based polymers are, however, typically non-degradable [33]. Another approach in biomaterial engineering is the composition of gelatin-based hydrogels containing variable amounts of lysine diisocyanate ethyl ester [34]. Their physicochemical properties and degradation behavior can be directly modulated by network formation. That makes them meet most of the criteria defined for good blood and biocompatibility of coating materials for cardiovascular implants [34,35]. Moreover, an in vitro and in vivo study demonstrates low inflammatory response and the absence of toxic effects in the spleen, liver, or kidney for hydrogels prepared with higher amounts of lysine diisocyanate ethyl ester [34]. A novelty is the incorporation of nanoparticles into hydrogels to strengthen the network and enhance their physicochemical and biological advantages [36]. In a recent study, Apte et al. show that Fe_3_O_4_ nanoparticle incorporation into agarose-based hydrogels modulates the response of platelets to the artificial surface and inhibits platelet adhesion and activation [37]. They found properties such as stiffness, adhesion force, and wettability—factors which will be discussed below—responsible for the bio-inert characteristics of their nanocomposite hydrogels.

## 3. Complement-Coagulation Interplay

**Figure 2:**
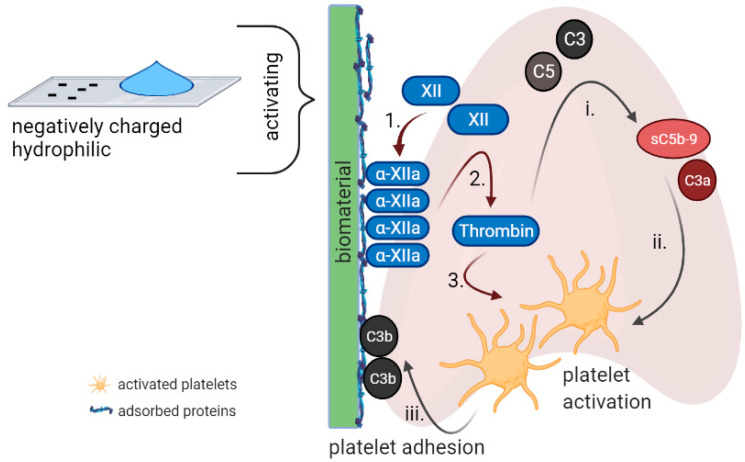
Surface-mediated complement-coagulation interplay. Factor XII of the contact system preferentially binds to negatively charged/hydrophilic surfaces generating Factor α-XIIa, which is also adsorbed onto biomaterial surfaces (1.) and further activates the contact system pathway of the coagulation cascade, generating thrombin (2.) that can, in turn, intensively activate platelets (3.). Thrombin cleaves C3 and C5 to C3a/C3b and C5a/sC5b-9, respectively, thus amplifying the activation of complement (i.) C3a activates platelets, enhancing their aggregation and adhesion. sC5b-9 is incorporated into the cellular membrane of platelets, inducing an alteration in membrane polarization and, thus, increasing the surface area on which clotting can occur (ii.). C3b binds to P-selectin and enhances platelet adhesion (iii.). Created with BioRender.com.

Biomaterial-induced thrombosis is usually attributed to blood coagulation initiated by the contact phase and platelet-related reactions to adsorbed plasma proteins [38]. For instance, even low levels of fibrinogen adsorption make the biomaterial platelet adhesive [39,40]. However, apart from the traditional role of platelets as mediators of hemostasis, there is evidence that platelet activation during thrombotic events is closely associated with the activation of complement eventually leading to “thrombo-inflammation” [8]. Plasma proteins such as fibrinogen, fibronectin, and vitronectin are prone to bind to biomaterial surfaces and are potent mediators of platelet adhesion [41,42]. In addition, Factor α-XIIa generated from auto-activation of FXII and adsorbed onto biomaterial surfaces activates the contact system pathway of the coagulation cascade, generating thrombin that can, in turn, intensively activate platelets [43,44,45] and cleave fibrinogen into fibrin [2,42]. Thereby, contact cascade is most efficiently activated in contact with negatively charged and/or hydrophilic surfaces [46]. Additionally, proteins of the complement system, which become activated upon contact with the biomaterial, support platelet adhesion and activation by directly enhancing blood clotting properties and by augmenting the inflammatory response, which, in turn, potentiates coagulation [42] —summarized in Figure 2. Once activated, platelets release chondroitin sulfate A from α-granules immobilizing various complement regulators on the platelet surface. Thus, activated platelets serve as ligands for the tethering of immune cells [47,48,49]. These findings underline that the two cascades, which have long been discussed as two entities, appear to modulate each other’s activity significantly making the appropriate design of a biomaterial even more difficult.

Complement and coagulation cascades act locally—complement is activated at the site of infection and coagulation at the site of bleeding. In general, biomaterial surfaces lack complement or coagulation regulators. Once the cascades are activated systemically as a result of contact to a biomaterial, they can become disorganized, leading to the accumulation of anaphylatoxins (C3a/b and C5a/b), the activation of immune cells, and the formation of thrombi which could seriously threaten the host [8]. In this context, Ekdahl et al. summarized that artificial surfaces that come into contact with blood preferentially activate either the complement system or the contact system [8,50,51]. In this context, graphene is discussed as a novel carbon-based material with unique crystal nanostructure and physical properties [52]. Improved blood compatibility compared to currently used materials facilitates the potential use of graphene coatings for cardiovascular implants [53,54,55]. A very recent study demonstrates that graphene coating reduced the activation of blood coagulation cascade in vitro and ex vivo in a rabbit model but did, however, not reduce complement activation [56].

Besides polymer coatings, biodegradable metals are under evaluation for cardiovascular implants [57]. Very recently, the blood compatibility of a Zinc-based alloy was determined using human blood. This study considered aspects of coagulation and complement activation. Further, the alloy also showed sufficient performance in vivo [58]. It should be noted, however, that a potential implant is not introduced into a healthy organism. Pre-existing cardiovascular diseases and risk factors may already activate the patient’s immune system. In vitro experiments with the blood of corresponding patients seem appropriate in this context.

## 4. Modulation of Complement Activation by Biomaterials

The design of biomaterials has been especially dedicated to the development of inert biomaterials, with the aim of limiting adverse reactions. Although inert biomaterials remain practically unchanged and tolerated by the host, regulation of cell adhesion to the protein layer adsorbed to biomaterials may change cell responses leading to improved wound healing [59,60]. Efforts to modulate the host response to biomaterials have included both chemical and physical approaches. Chemical approaches focus on preventing complement and coagulation activation through the reduction of protein adsorption or the incorporation of pharmacological agents. On the other hand, physical approaches include the modulation of surface topography and mechanical properties of a biomaterial. Besides these strategies to overcome adverse effects of biomaterials, an emerging approach is the incorporation of endogenously expressed biomolecules that naturally modulate the host response to the biomaterial. Immobilization of growth factors, such as VEGF, TGF-β, and PDGF, control adhesion, migration, proliferation, and differentiation of various cell types and provide an anti-thrombotic environment [60,61]. Although some materials are capable of reducing protein adsorption and/or cell adhesion, there is currently no material available that diminishes biomaterial associated thrombo-inflammation.

### 4.1. Surface Chemistry

The interaction of a biomaterial with the adsorbed protein layer is crucial for the body reaction to an implant. In this context, surface wettability is an important factor affecting the initial adsorption of blood proteins. Here, hydrophilic—wettable—surfaces are mostly associated with low surface interactions with blood components. Various approaches aim to create less adhesive surfaces to control the amount, composition, and conformational changes of bound proteins [4]. The immune system recognizes hydrophobic parts of biological molecules as universal damage-associated molecular patterns and subsequently triggers the processes that lead to their elimination [4]. Thus, it is obvious that hydrophobic surfaces are more prone to protein adsorption than hydrophilic surfaces and that proteins show little adsorption to hydrophilic surfaces which might be due to the preservation of their native state secondary structure [62,63,64]. In this context, numerous studies report that white blood cell activation is controlled by surface wettability and dependent on the cell type [65,66]. While macrophages and lymphocytes are preferentially activated by hydrophilic/anionic surfaces, hydrophobic surfaces are selective for CD8 T lymphocytes.

Another important surface property is the surface charge, which is determined by the presence of chemical groups such as positively charged amino (-NH_2_), negatively charged carboxyl (-COOH), and neutral hydroxyl (-OH) and methyl (-CH_3_) groups. Thereby, hydrophilic amino and hydroxyl groups induce the highest infiltration of inflammatory cells in vivo [4,67], which might be explained by the ability of C3b to covalently bind hydroxyl groups [25,68]. Of note, platelets and biological surfaces are negatively charged and thus attracted to positively charged surfaces [69]. On the other hand, hydrophilic positively charged amino and negatively charged carboxyl groups both cause stronger conformational changes of adsorbed proteins and thus may trigger the contact system [70]. Pacharra et al. therefore observed stronger platelet adhesion to modified PCL films, which present negatively charged carboxyl groups on their surface. However, very low amounts of PCL copolymerized with PLLA were not sufficient to create a negatively charged surface and thus reduced platelet adhesion in this study [14]. Further studies applying self-assembled monolayers of graded hydrophilicity revealed significantly increased fibrinogen adsorption on hydrophobic methyl groups compared to hydrophilic carboxyl surfaces, with the same tendency for platelet adhesion [71]. Since the initial cell response is triggered rather by the adsorbed protein than by the surface itself, the pattern in which blood proteins adhere determine material-related cellular reactions. Unfortunately, modulating the surface chemistry is not enough to foresee the behavior of bound proteins on artificial surfaces and reported results are contradictory [4], depending on the study design and species used for experiments.

### 4.2. Topography

The surface of a biomaterial can be modified by different techniques such as particle deposition, self-assembled monolayers, soft photolithography, blasting, acid etching, and polymer expansion which finally result in different size geometries, surface protrusions, or dentations [4,72]. Thereby surface patterning modulates cellular and physiological processes such as the binding affinity of blood proteins [73,74] and thus host response to the foreign material [65]. Surface patterns, ranging between 10 and 100 nm, are usually utilized to directly modulate cell behavior since they directly change surface properties such as surface charge, energy, and topography and are reported to enhance cellular functions due to conformational changes of the adsorbed protein [74,75,76]. Increasing the nanoscale roughness from 15 nm to 30 nm, for example, induces a significant decrease in protein binding affinity [74]. Furthermore, in vitro data highlight the importance of nanoparticle size, charge, and architecture for subsequent complement activation as polymeric and spherical structures tend to damage the cell membrane of immune cells [77]. For example, platelet activation is generally reduced on nanostructured gold nanoparticles and is more sensitive to nanotopography than surface hydrophobicity [78]. In addition, nanoparticles carrying native complement receptors such as C5aR were able to block interactions between C5a and C5aR, thereby reducing neutrophil activation [79].

Overall, these data indicate that surface nanostructure and nano-scaled roughness are potentially relevant morphological features, which regulate protein adsorption and direct cellular response to biomaterials. However, the mechanism behind the topography-induced cellular response is complex and still unclear.

### 4.3. Surface Roughness and Stiffness

Surface roughness refers to the structure and topography of the top surface layer and is most commonly used to characterize a surface [80]. It is well established that cell functions are tightly related to the cellular interactions with the extracellular matrix. In particular, topographical features have the ability to target receptor-driven pathways and thus mediate the appropriate cellular responses [81]. In this context, matrix stiffness and surface roughness have been recognized as key factors directly regulating, for example, cell adhesion [82,83].

Polymeric and metallic implant surfaces are by nature rough at a cellular level, which accounts for the observed increased thrombogenicity of these materials [69]. With respect to activation of the coagulation cascade, a recent study demonstrates that increasing surface roughness positively correlates with the number of adhered platelets [84]. Most studies in this context are focused on surface roughness and are evaluated on only a few different roughness values due to the limited manufacturing technologies. Only recently, Hou et al. presented a high-throughput tool to study the influence of the combined surface roughness and substrate stiffness on cell adhesion and mechanotransduction of mesenchymal stem cells using soft and stiff hydrogels with integrated surface roughness gradient [82]. They clearly demonstrate the synergy of both surface characteristics and their direct influence on cell behavior. Scott et al. demonstrated that substrate stiffness of polyethylenglycol-based hydrogels directly impacts macrophage morphology, surface marker expression, and growth factor production [85]. These properties reflect the influence of matrix stiffness on macrophage polarization, which will be further specified below. Moreover, it has been shown that material stiffness influences the composition of the adsorbed protein layer and that tissue cells are able to sense material stiffness through this layer [86].

It is, however, difficult to precisely predict blood compatibility regarding protein adsorption, cell adhesion, and activation due to the variety of influencing parameters, as the processes during the contact of the blood with the biomaterial are complex.

### 4.4. Immune Modulation Strategies

Over the past decade, evidence has emerged that the immune system plays a critical role in controlling and determining the nature of the repair process and that the inflammatory response is not an undesirable reaction but an important component of tissue repair and regeneration [87]. Initiating very specific biological responses through biomaterial design could therefore be beneficial for both implant integration and performance [4]. By binding naturally expressed proteins which directly interact with receptors expressed by immune cells or indirectly modulate immune cells by regulating complement or coagulation cascade, biomaterials can directly influence the host response [23,65]. For example, peptides were immobilized on surfaces that bind factor H with high affinity, which led to a significant reduction in circulating C3a- and sC5b-9 in vitro [88].

Local tissue injury at the site of biomaterial implantation may promote neutrophil and macrophage recruitment through damage-associated molecular patterns, cytokines, and chemokines. Therefore, immunomodulatory biomaterials are targeted to promote desirable events, such as the polarization of M1 to M2 macrophages, which have anti-inflammatory/anti-fibrotic properties [89,90,91]. M2 macrophages, in turn, contribute to regeneration via crosstalk with regulatory T cells, which promote a pro-regenerative tissue repair cascade [92]. Impairment in the M1-to-M2 transition, such as prolonged M1 macrophage phenotype, has been implicated in myocardial infarction [93]. Efforts to influence the immune response to biomaterials include both chemical and physical approaches modulating macrophage phenotype. These strategies can prevent initial protein adsorption to diminish downstream activation of complement and coagulation [94]. However, protein adsorption might not be solely responsible for macrophage activation, since neutral or hydrophilic surfaces, which exhibit less protein adsorption, induced the secretion of inflammatory cytokines [95]. The addition of anti-inflammatory agents such as heparin directs the adaptive immune response by switching from Th1 to Th2 lymphocytes, rapidly reducing inflammation to promote wound healing [4,96,97].

The mechanisms of material-induced leukocyte activation are still not understood, which hampers the development of biomaterials and delivery systems that can modulate the immune system. Inhibitor studies targeting either the complement system at C3 level or interfering with blood coagulation suggest that both processes play a crucial role [8,23,98,99]. Since most approaches only affect one of the players in the host’s response to the biomaterial, no single inhibitor has yet been identified that could be sufficient to provide clinical benefits. Consequently, the requirements for an ideal biomaterial change from “immune-evasive”—aimed to reduce host responses—to “immune-interactive” enabling the desired immunological responses for successful integration of the biomaterial and subsequent tissue repair [4].

## 5. In Vitro Testing

Since the concept of blood compatibility is not properly defined, various standards concerning different aspects have been proposed for the biological performance of materials. Most commonly, such approaches track the ability of materials or coatings to resist non-specific protein adsorption [100] and/or platelet adhesion [101] or to reduce the degree of platelet and complement activation [7,102]. All three aspects will eventually influence the occurrence of thrombosis and inflammation because they are highly linked [17,103,104]. The success of the resulting therapeutic strategies will depend on the extent to which biological processes are modulated and how current medication influences this regulation [7,105,106,107]. Systemic studies addressing these questions are hardly done by only one laboratory and are thus rare. Importantly, the preparation of the materials and the blood samples is an integral requirement for standardized and reproducible in vitro testing strategies [108]. Thereby, special attention is paid to the importance of the availability of endotoxin-free and thoroughly (physically and chemically) characterized materials since bacterial endotoxins activate blood cells.

**Figure 3:**
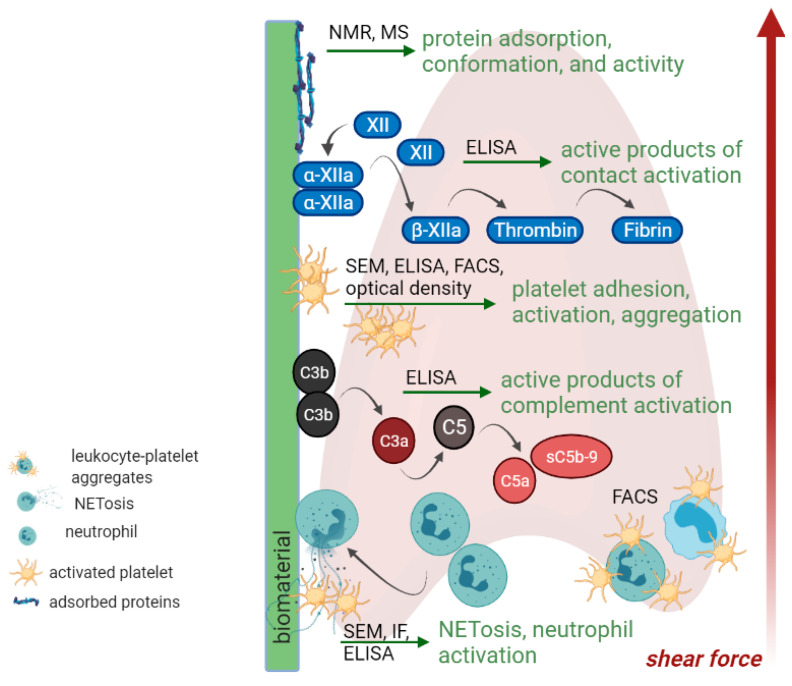
In vitro testing strategies: overview of targets and techniques to assess blood compatibility of biomaterials. Created with BioRender.com.

Which parameters should be analyzed in vitro to predict in vivo performance of a biomaterial in contact with blood? *ISO 10993-4:2017* [109] recommends tests for hemolysis, coagulation, platelet, complement and leukocyte activation as an initial guide to blood compatibility testing. However, other aspects should also be considered: the analysis of surface adsorbed proteins, the establishment of appropriate control systems, the consideration of blood collection procedures for in vitro tests, and the choice of anticoagulants [110,111]. Figure 3 gives an overview of possible targets of blood compatibility testing. *ISO 10993-4:2017* provides, however, only recommendations on the in vitro test strategy. Standardized protocols or standard operation procedures, agreement on reference materials and experimental conditions (static or dynamic, flow conditions, test duration depending on the biological processes), and, very importantly, the specification of cell models are still lacking [108]. For example, the use of blood products or animal blood should be avoided, due to functional alterations [112] and species-dependent platelet function [113,114], respectively. Here, Braune et al. recommend using freshly drawn blood from healthy donors that should be characterized comprehensively [108,115].

### 5.1. Protein Adsorption

The vast interactions of the blood with the artificial surface are determined by its geometry, the shear forces that occur at the blood-contacting surface, and the physical and chemical properties. In this context, the adsorption of plasma proteins is the initial event and the basis for all subsequent interactions [105,116,117]. Highly mobile proteins quickly adsorb onto the biomaterial and are subsequently replaced by proteins with a higher affinity called the Vroman effect [118]. The composition of the adsorbed protein layer and the conformation of the proteins are unique for each biomaterial [50]. The methods applied to detect surface adsorbed proteins are carried out under static conditions and mainly include spectroscopic and/or mass spectrometric approaches [108,119]. However, the precise prediction of the dynamics within the protein layer remains challenging [120].

The conformational changes of adsorbed proteins must also be considered in this context. They directly affect the interactions with blood cells by exposing binding motifs that are not accessible to the corresponding receptors and enzymatically active blood components in the native state [121,122]. The unfolding of fibrinogen, for example, exhibits platelet binding sites at the fibrinogen γ- and α-chains. It has been reported that the degree of unfolding correlates with the activation and adhesion of platelets to artificial surfaces and might be unrelated to the actual amount of adsorbed fibrinogen [70]. This makes fibrinogen likely as the critical ligand for adhesion. The conformation and bioactivity of surface-bound proteins can be accessed via spectroscopic and microscopic approaches [117,121].

### 5.2. Contact System, Coagulation Cascade and Platelet Activation

Blood-contacting medical devices may cause thrombo-inflammation due to changes in physiologic blood flow patterns and contact with foreign materials. The intrinsic coagulation pathway is initiated by Factor XII—the main circulating zymogen of the contact system—which recognizes the artificial surface [46,123]. Reports on FXII-mediated interactions demonstrate the impact of negatively charged surfaces, as well as highly wettable and highly non-wettable surfaces [43]. Interactions with such surfaces result in conformational changes of FXII, which eventually leads to its auto-activation by conversion into α-FXIIa [43,124]. The immobilized α-FXIIa cleaves circulating FXII to enzymatically active β-FXIIa, which subsequently converges in thrombin generation and thrombus formation [46,123]. Typically, ELISA techniques are applied to measure the various active products of contact activation and thrombin generation. Further, microscopy is a common tool for the analysis of adherent and activated platelets on biomaterials. Conventional methods only take into account small sections of the entire surface and therefore lack statistical validity and standardization [108]. Clauser et al. applied image segmentation and machine learning algorithms to automatically analyze over 100,000 microscopic images proposing a reliable, comparable, and standardized approach to determine platelet adhesion and activation on biomaterials [125].

Platelets respond to minimal stimulation and become activated when they contact any thrombogenic surfaces, such as injured endothelium or artificial surfaces or when flow patterns are changed. The effect of shear forces on platelets has been studied extensively. Higher shear rates result in higher platelet deposition, while at lower shear rates the inverse is true [126,127]. In vitro tests under dynamic conditions differ greatly in their design and flow dynamics and should, regarding cardiovascular biomaterials, consider both physiological arterial conditions (pulsatile flow with shear rates >1500 s^−1^) and pathological conditions (supra-physiological shear rates >10,000 s^−1^, turbulent flow, and recirculation) [16,128,129,130,131]. In this context thrombin generation and platelet activation are both highly dependent on the shear rate. Further, the degree of platelet activation correlates with both the shear stress magnitude and exposure time [132]. Microfluidic devices provide an easily adjustable platform to expose cells, proteins, platelets, or whole blood to flow and shear stress. Such technologies can be easily adapted to specific questions and represent a significant advance for studying the effects of shear forces on biological processes [132,133].

However, there is currently no standardized in vitro test protocol to evaluate device thrombogenicity [134]. Addressing this problem, Braune et al. conducted a unique prospective, randomized, and double-blind multicenter study demonstrating that standardization of in vitro test protocols allows a reproducible assessment of platelet adhesion and activation from fresh human platelet-rich plasma [135]. In five independent German test centers, platelet density, platelet covered surface area, and area per platelet were determined on three different polymers using a stringently standardized in vitro test protocol. The remarkable results show that a reproducible evaluation of the adhesion and activation of human platelets on polymer-based biomaterials is possible. Although blood donors (*n* = 10 for each center) were not age- and/or gender-matched and processes, such as blood preparation, were not harmonized, the scoring for the thrombogenic potential of the materials was equal for all participating centers. This is an important aspect that should be considered by the scientific community, in the future.

### 5.3. Complement and Leukocyte Activation

Human blood contains 4.3–10 × 10^3^ leukocytes/μL, such as granulocytes, lymphocytes, and monocytes. Monocytes make up only 1–6% of all leukocytes, and neutrophilic granulocytes are the most abundant at 50–70%. When foreign material is detected, these immune cells are rapidly activated by the complement system. Products of the complement cascade lead to an increased permeability of blood vessels, attract and activate neutrophils and monocytes and thus stimulate the release of Tissue Factor, which initiates the coagulation cascade [23]. In endothelial cells, products of the complement cascade lead to increased expression of cytokines, chemokines, and adhesion molecules [99,136]. To determine complement activation, *ISO 10993–4:2017* recommends the in vitro determination of C3a, C5a, and sC5b-9 by ELISA assay. Moreover, Engberg et al. demonstrated a direct correlation between downstream biological effects and the proteins initially adhering to an artificial surface after contact with blood [50]. They found strong correlations between the ratio of C4 to its inhibitor C4BP. However, the levels of complement activation products C3a and C5a/sC5b-9 correlated only weakly or not at all, questioning their predictive value.

While the importance of flow has been recognized, our current understanding of its mechanisms is focused mostly on platelets. Interestingly, platelets can initiate complement activation by generating C5b-9 proportional to increasing shear stress and exposure time [103,137]. Further, physiological shear stress antagonizes the activation of complement and coagulation cascade via the expression of the inhibitory proteins in endothelial cells [138,139]. In contrast, non-physiological flow patterns (turbulent and oscillatory flow) induce endothelial expression of high amounts of properdin, a known activator of the alternative pathway of the complement cascade [140]. Finally, neutrophilic granulocytes contribute to the activation of the complement cascade via NETosis. NETosis is a host defense mechanism involving the extrusion of DNA-rich nucleic material, histones, and enzymes, so-called neutrophil extracellular traps (NETs) [141]. Originally, NETosis was described as a mechanism to clear pathogens from the blood [142]. However, shear-induced NETosis promotes complement activation as well as products of complement activation stimulate NETosis, eventually cumulating in thrombo-inflammation [143,144]. The impact, which biomaterials might have on NETosis, is rarely the subject of investigations. Only one study demonstrates that neutrophils are sensitive to changes in biomaterial surface properties and exhibit differential activation in response to a titanium surface [145].

In vivo, contact with cardiovascular devices activates both neutrophils and monocytes. More recently, Witherel et al. presented an in vitro model for macrophage interaction with biomaterials that claimed to apply to a wide range of biomaterials [92]. There is an imminent need for comparable in vitro models to overcome the lack of standardized operating procedures for blood cell activation.

### 5.4. Leukocyte-Platelet Aggregates

Activated leukocytes contribute to thrombosis through pro-coagulant properties such as the formation of leukocyte-platelet aggregates [38]. Such interactions are a new aspect in the study of blood compatibility. However, the effects of this association are largely unknown. For example, leukocyte-platelet aggregates could contribute directly or indirectly to thrombin generation via monocyte tissue factor [9].

In this context, the effect of shear stress on blood cell activation is well known. It is assumed that physiological shear rates cannot activate leukocytes [16,146]. The presence of a biomaterial might very well be a potential stimulus for blood cells triggering the formation of leukocyte-platelet aggregates even under physiological shear rates. Recent data of our group show that the presence of PLLA induces more circulating monocyte-platelet aggregates under flow conditions, which is accompanied by an enhanced platelet and monocyte activation [16]. Chang also observed significant material-induced leukocyte-platelet aggregation, and tissue factor expression in response to pathological shear rates [147]. Overall, we show a complex interaction of hemodynamic forces and the underlying polymers regarding blood cell activation and leukocyte-platelet interaction which is possibly influenced by surface wettability and protein adsorption. Circulating leukocyte-platelet aggregates are frequently analyzed and quantified by flow cytometry. Special attention must be paid to the selected surface markers, depending on which subpopulation is to be considered [16,148].

Data of clinical trials prove the involvement of leukocytes and cytokine secretion in thrombotic complications [149], and in vitro studies suggest that the intrinsic pathway alone might not be primarily responsible for platelet activation [9,38]. In the context of blood compatibility testing, leukocyte adhesion/activation and formation of platelet-leukocyte aggregates might thus possess a stronger predictive power than platelet activation alone [16].

### 5.5. Proteomic Approaches

Proteomics-based approaches are particularly well suited to provide a more comprehensive picture of biological processes by examining the entire proteome of a biological environment rather than each protein individually and are thoroughly reviewed in [119,150]. Understanding the global cellular and molecular context of the interactions of biological systems with biomaterials may lead to a better prediction of material behavior from in vitro to in vivo.

It is commonly accepted that initial protein adsorption determines the performance of a biomaterial [151]. Proteins adsorbed to the material surface facilitate and control cell adhesion and the subsequent cellular events, including cell proliferation and differentiation [152]. The quantity and properties of the adsorbed proteins are highly dependent on the surface properties [121]. The first layer of attached proteins, thereby, determines the formation and content of subsequent protein layers, affecting the fate of the material itself, in terms of blood compatibility and degradation [150]. Particularly competitive protein adsorption to a biomaterial is an important issue, which cannot be addressed properly by commonly used techniques (see Section 5.1) [119]. Using proteomics it is now possible to characterize the composition of an adsorbed protein layer regarding the identity of different proteins and their concentration in a complex biological system [153]. Milleret et al. highlighted the importance of surface properties by demonstrating that neutrophil adhesion to a cobalt chromium alloy pre-coated with fibrinogen is mediated by protein orientation and conformation rather than the amount of adsorbed protein [154]. Regarding plasma protein, fibrinogen, albumin, and immunoglobulin γ are the most frequently studied proteins due to their high abundance [39]. However, proteomic analyses identified other low abundance plasma proteins such as serum amyloid P [155], complement components [50,156] as a potential mediator of implant failure. Moreover, Swartzlander et al. identified 245 inflammatory proteins (out of 300 identified proteins) adsorbed on poly-ethylene glycol hydrogels [157]. In a recent study, Ndumiso et al. compared the protein corona of PLGA and PCL nanoparticles incubated with human serum and demonstrated that each biomaterial shows a unique proteome fingerprint, which is influenced by surface characteristics such as wettability and by thermodynamics of protein binding [158]. Regarding the foreign body response, Buck et al. demonstrated that polystyrene surfaces containing carboxyl groups adsorbed more proteins associated with pathways that are involved in wound healing and implant integration than, for example, surfaces containing amino groups [159]. It seems evident that proteomics is a powerful approach to assess protein-binding dynamics and the composition of adsorbed monolayers [119,150]. However, it needs to be complemented with other state-of-the-art analytical tools to complete the understanding of host biomaterial interactions.

## 6. Concluding Remarks and Open Questions

The complexity of the interactions between blood and material explains why it has not yet been possible to develop truly blood compatible materials. Currently, we are far from describing a complete mechanism of the material-induced host response. Simplifying the system neglects many aspects of the complex material-blood interaction and has not yielded any real progress. The passivation of an artificial surface intends to minimize protein adsorption since cells interact with pre-adsorbed proteins and not with the bare surface. Despite large efforts in developing non-activating stent coatings, the host responses and subsequent blood cell activation and adhesion lead to incompatibility reactions often limiting the functionality of a device. To overcome unfavorable material-related biological responses surface modification strategies that enable biological processes (bioactive coatings) are designed to regulate complement and coagulation activation. However, newly developed designs have to be tested thoroughly in vitro in a more strategic and standardized fashion than in the past. Moreover, expertise on the individual procedures and techniques should be pooled, as comprehensive in vitro testing cannot be performed in a single laboratory. Since future blood compatible surfaces will target much more specifically the intended biological responses and suppress undesired cellular and molecular reactions, they require a detailed understanding of the physiological activation pathways and their intercorrelations. The use of more physiological experimental designs should result in significant advances in our knowledge on the effect of mechanical factors on thrombosis and hemostasis.
